# Proximate body composition, elemental concentration and enzymatic activity analysis of *Catla catla* exposed to the nanoparticle (CuO-NPs) and Vitamin C

**DOI:** 10.1371/journal.pone.0339412

**Published:** 2026-02-12

**Authors:** Najeeb Ur Rehman, Syed Qaswar Ali Shah, Muhammad Naeem, Huma Naz, Muhammad Rafiq

**Affiliations:** 1 Cholistan Institute of Biological Sciences, Cholistan University of Veterinary and Animal Science, Bahawalpur, Pakistan; 2 Institute of Zoology, Bahauddin Zakariya University, Multan, Pakistan; 3 Department of Physiology and Biochemistry, Cholistan University of Veterinary and Animal Science, Bahawalpur, Pakistan; Tanta University Faculty of Agriculture, EGYPT

## Abstract

Aquaculture plays a vital role in global food production, providing a significant source of protein and livelihood for millions of people worldwide. The toxicity of copper oxide nanoparticle (CuO-NPs) can severely impact the health and productivity of farmed fish species, such as *Catla catla*. This research aims to evaluate the effectiveness of Vitamin C in protecting *C. catla* from the harmful effects of CuO-NPs, thereby contributing to sustainable aquaculture practices. Fish exposed to various levels of CuO-NPs and Vitamin C exhibited varying degrees of responses for proximate body composition, elemental concentration, and digestive enzymes. A total of 540 samples of *C. catla* were collected from Bahawalpur Fish Hatchery, Punjab, Pakistan. CuO-NPs synthesized by green synthesis from neem extracts (*Azadirachta indica*) added in fish feed and exposed for 90 days in glass aquaria (Triplicate) with five experimental groups as T-I (2 mg/kg CuO-NPs), T-II (4 mg/kg CuO-NPs), T-III (2 mg/kg CuO-NPs with 250 mg/kg Vitamin C), T-IV (4 mg/kg CuO-NPs with 250 mg/kg Vitamin C) and T-V (250 mg/kg Vitamin C) and one control group (T-O). Results indicates that mean percent (%) moisture content was found 76.72 ± 1.82, 77.31 ± 2.63, 78.92 ± 2.12, 77.89 ± 1.09, 78.11 ± 1.45 and 76.50 ± 1.56 for T-O, T-I, T-II, T-III, T-IV and T-V, respectively. Lowest mean value of percent moisture content was found in T-V, while highest value was noted in T-II. The highest mean protein content (%) in *C. catla* was found in T-V (Vitamin C) while lowest in T-II (CuO-NPs 4 mg/kg). Concentrations of cadmium, lead and cobalt were non-quantifiable among all treatment groups, while the concentration (µgg^-1^) of all other studied metals (Fe, K, Na, Zn, Cr and Cu) found below the permissible limits. Regression analysis used to examine how different treatments affected the amounts of different elements in different studied groups. The levels of Fe, K, Na, and Zn were found higher in CuO-NPs treatments (T-I, T-II) compare with Vitamin C (T-III, T-IV), while the levels were levels found higher in T-V and highest in control group. Activities of digestive enzymes in various groups showed a significant difference with maximum value of protease activity in T-V (5.734 ± 0.60 U/mL.min^-1^), while lower in T-II (4.68 ± 0.78). Activities of amylase was found maximum value in T-V (0.74 ± 0.14 U/mL.min^-1^), while lower in T-V (0.54 ± 0.19). Maximum activities of lipase activity were observed in T-0, while lower in T-II.

## Introduction

Aquaculture produces a cheap source of animal protein for human. In the past few years, there has been a remarkable increase in fulfilling food demands, which also enhances the living standard of people and supports the national economy [1]. Generally, fish comprise 66% to 81% water, 16% to 21% protein, 1.2% to 1.5% mineral, 0.2% to 25% fat, and 0% to 0.5% carbohydrate [2]. Carbohydrates and non-protein components are often disregarded for regular analysis due to their small presence [3,4]. The water content in a fish’s body is a good indicator of its protein and fat levels. It is inversely related to the amounts of lipids and proteins, meaning that as the water percentage decreases, the levels of lipids and proteins increase, resulting in higher energy content in the fish [5].

Metal bioaccumulation in the aquatic ecosystem directly affect the physiological system of organisms [6]. The entry of heavy metals into the body of aquatic organisms may be restricted due to their larger size. However, the nanosized particles can quickly enter the fish body through inhalation and ingestion. Studies reveal the nature of particles and the damages caused by the bioaccumulation of nanoparticles in aquatic organisms. Insufficient data is available on the nature of toxicity and mechanism of action of nanoparticles on the physical system of fishes. The marine organisms obtain the source of chemicals and accumulate them through contact with water or sediments and by ingesting contaminated food. The bioaccumulation of toxic substances in the tissues and organs of fish is a warning sign for the potentially toxic impact on the health of human life [7].

Animal growth depends on its feed and nutrient profile and ability to ingest, digest, absorb, and metabolize feed nutrients [8]. Different fishes’ digestive enzyme activity depends on age and development [9]. Comparing digestive enzyme concentrations (lipase, amylase, and trypsin) to gut mass, gut length (GL), and Zihler’s index (ZI) was necessary. Thus, fish relative gut mass (RGM) and ZI varied progressively with body mass [10]. Lipase, amylase and protease concentrations increase with body weight [11]. Digestive enzyme activity provides nutrient- and cost-effective feed formulations for different fish sizes [12]. The digestive tract enzyme content affects nutrition digestion [8]. Gut digestive enzyme concentration depends on eating and gut architecture. In vertebrates, energy metabolism is the most crucial physiological function. Their energy needs are met by digesting organic food (proteins, lipids, and carbohydrate). The availability of nutrients for all biological activities depends on fish metabolism digesting [13].

The aim of this study was to study proximate composition, elemental concentrations and digestive enzymes of *Catla catla* exposing with various concentrations of CuO nanoparticles and vitamin C.

## Materials and methods

### Ethics statement

Institutional review board statement. It has been confirmed that the experimental data collection complied with appropriate permissions from Ethical Review Committee, Department of Zoology, Cholistan University of Veterinary and Animal Science, Bahawalpur, Pakistan. The study did not involve humans.

### Sampling

Five hundred and forty (540) *C. catla* were collected from the Bahawalpur Fish Hatchery, Punjab, Pakistan. These samples were packed and transported in oxygen-filled containers at Fish Laboratory, Bio-Park, Bahauddin Zakariya University Multan Pakistan (Latitude 29°23’60.00“N; Longitude 71°40’59.99” E) and acclimatized in aquaria for 15 days under laboratory conditions.

### Experimental design

**Fig 1:**
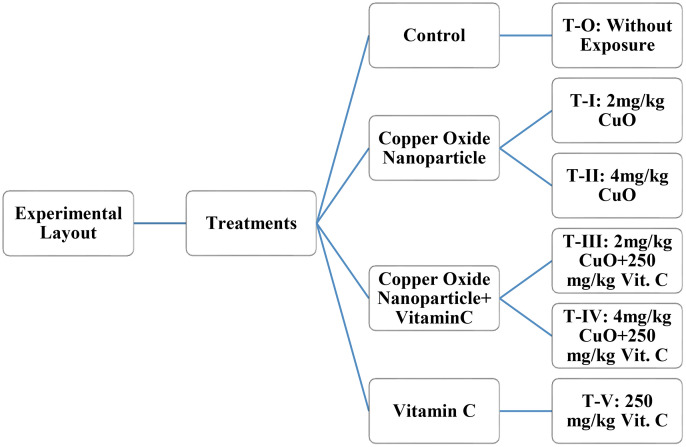
Experimental Layout exposed to different concentration in *C. catla.*

After acclimatization, an experimental trial was conducted for 90 days in glass aquaria (40*40*40 cm). Thirty (30) Catla catla were stocked in each aquarium, with treatments conducted in triplicate across six groups (Fig 1): a control group T-O (without exposure), T-I (2 mg/kg CuO-NPs), T-II (4 mg/kg CuO-NPs), T-III (2 mg/kg CuO-NPs + 250 mg/kg Vitamin C), T-IV (4 mg/kg CuO-NPs + 250 mg/kg Vitamin C) and T-V (250 mg/kg Vitamin C), in which fish fed with a diet containing 25% crude protein as a basal diet, two times a day with a total of 3% of body weight of the fish. At the end of the trial, fish specimens were immersed and kept in solution of MS222 (250 mg/L) for 10 minutes to euthanize the fish.

### Proximate body composition

For comparative analysis of proximate body composition in different concentration of nanoparticle and vitamins, 10 fishes from each treatment and a total of 60 *C. catla* (8.89 ± 2.05 g) were used to access the moisture, ash, fat, protein and organic content by using standard methods [14]

Each fish was dried, until constantly weighed, in a pre-weigh aluminum tray in an electric oven at 70–80°C to determine moisture content.

Total ash content of the fish was determined by burning dry powder of sample at 550^o^C for 24 h in muffle furnace (RJM-1.8-10A). The incinerated samples were weighed for ash determination, after cooling in the desiccator.**L**ipid content was determined by a dry extraction method in which a mixture of 1:2 chloroform and methanol method.

Protein content of the fish samples was assessed by subtracting it from the weight of other leading contents, i.e., moisture, ash and fat, following Salam and Davies [14].

### Elemental composition

The same samples of *C. catla* used for proximate analysis were further processed and utilized for elemental analysis. The white ash of each sample, obtained during proximate analysis, was dissolved in a conical flask with 10 mL (70%) HNO_3_, placed on a hot plate at 82–100°C, heated to dryness, and diluted up to 25 mL with deionized water. The concentrations of different elements (Cu, K, Na, Cd, Cr, Pb, Co, Zn and Fe were determined by running the sample solution through an atomic absorption Spectrometer (Model: Agilent 240AA) [15].

### Digestive enzymes

After the completion of the experimental trial, ten fish samples (from each treatment were randomly collected and size was taken to study the digestive enzymes. To safely extract digestive enzymes (lipases, proteases, and amylases), the entire intestine was removed, weighted (in grams) and length was measured (in cm). Relative gut mass (g), relative gut length (cm) and Zihler’s Index (ZI) was calculated. Each gut sample was wrapped in aluminum foil, and frozen at 10^o^C. These samples were taken in a micro centrifuge tube and homogenized in a homogenizer using cold Tris-HCl. The homogenate was centrifuged for 15 minutes at 4^o^C at 6000 × g, and the supernatant was collected and kept at 0^o^C to estimate the enzyme concentration. The concentrations of amylase, protease, and lipase were determined by following Riaz and Naeem [9].

### Statistical data analysis

One-way ANOVA followed by Duncan’s multiple range test was applied to determine significant differences (P < 0.05) in mean values among different experimental groups in proximate and elemental composition data. Linear regression analysis and calculation of correlation coefficients were made to observe the variations in body composition and elemental concentrations in relation to body weight and total length of *C. catla* [15]. For regression analyses, P value ,0.05 considered as least significant, 0.01 significant while P value <0.001 considered as highly significant correlation [16].

## Results

Mean values of percent (%) moisture, ash, fat, protein, organic content and fat free mass in wet (ww) and dry body weight (dw) of *C. catla* are shown in Table 1. In the present study, percent moisture content in all studied groups were ranged from 73.78 to 79.82% with the significant highest (P < 0.05) mean value in T-II. Highest mean value of ash content (ww) in *C. catla* samples was found in T-O and T-II, which were not significantly different (P < 0.05) from each other. Percent fat content ranged from 3.80% to 4.53% in the wet weight but no significant difference (P > 0.05) was found among different groups. The highest protein content (P < 0.05) in wet and dry body weight of *C. catla* was found in T-V in which the fish were fed vitamin C with the basal diet.

**Table 1:** Ranges and mean values of different body constituents in percentage of wet (WW) and dry weight (DW) for *C. catla.*

Constituents		T-O (Control)		T-I (CuO 2 mg)		T-II (CuO 4 mg)			T-III (CuO 2 mg + Vit 250 mg)		T-IV (CuO 4 mg + Vit 250 mg)		T-V (Vit 250 mg)	Sig.
		Range	Mean±S.D.	Range	Mean±S.D.	Range	Mean±S.D.		Range	Mean±S.D.	Range	Mean±S.D.	Range	Mean±S.D.
Moisture %		73.78-79.82	76.72 **±** 1.82^b^	72.94**-**80.67	77.31 **±** 2.63^ab^	76.13-83.87	78.92 **±** 2.12^a^	75.42-78.88	77.89 **±** 1.09^ab^	75.31-79.89	78.11 **±** 1.45^ab^	73.81-78.89	76.50 **±** 1.56^b^	0.46
Ash	WW%	2.80-5.54	4.01 **±** 0.87^a^	3.18-4.44	3.84 **±** 0.45^ab^	2.61-4.79	4.01 **±** 0.76^a^	3.16-3.64	3.39 **±** 0.13^b^	3.25-5.00	3.80 **±** 0.59^ab^	2.24-2.94	2.66 **±** 0.25^c^	0.000
	DW%	11.94-27.43	17.49 **±** 4.95^ab^	16.13-18.25	16.91 **±** 0.65^ab^	12.00-28.27	19.24 **±** 4.51^a^	13.37-16.50	15.36 **±** 1.00^b^	14.38-23.62	17.40 **±** 2.93^ab^	9.41-13.95	11.39 **±** 1.51^c^	0.000
Fat	WW%	3.51-5.79	4.53 **±** 0.78	2.94-5.98	4.11 **±** 1.00	2.58-5.68	4.06 **±** 0.97	2.65-5.07	3.80 **±** 0.77	3.10-5.25	3.86 **±** 0.66	2.69-4.80	4.00 **±** 0.80	0.457
	DW%	13.39-27.55	19.72 **±** 4.51	11.00-22.54	18.10 **±** 3.49	14.00-26.45	19.18 **±** 3.83	12.00-23.00	17.20 **±** 3.36	15.40-24.35	17.64 **±** 2.94	11.29-21.00	17.04 **±** 3.20	0.476
Protein	WW%	10.16-19.45	14.75 **±** 3.06^b^	13.06-19.41	14.74 **±** 1.95^b^	8.99-15.80	13.01 **±** 1.98^b^	13.05-16.87	14.92 **±** 1.16^b^	12.64-17.16	14.24 **±** 1.47^b^	14.37-19.05	16.83 **±** 1.68^a^	0.004
	DW%	50.34-74.19	62.79 **±** 8.75^bc^	60.20-72.71	64.99 **±** 3.77^bc^	51.27-68.00	61.58 **±** 5.68^c^	61.47-72.06	67.44 **±** 3.41^ab^	59.72-69.50	64.96 **±** 3.76^bc^	67.00-79.31	71.56 **±** 4.28^a^	0.001
Organic content	WW%	14.64-22.96	19.28 **±** 2.48^ab^	16.15-22.69	18.85 **±** 2.22^bc^	11.57-19.16	17.07 **±** 2.26^c^	17.75-21.29	18.72 **±** 1.12^bc^	16.17-21.14	18.09 **±** 1.55^bc^	18.17-23.68	20.83 **±** 1.51^a^	0.003
	DW%	72.57-88.06	82.51 **±** 4.95^bc^	81.75-83.87	83.09 **±** 0.65^bc^	71.73-88.00	80.76 **±** 4.51^c^	83.50-86.63	84.64 **±** 1.00^b^	76.38-85.62	82.60 **±** 2.93^bc^	86.05-90.59	88.61 **±** 1.51^a^	0.000
Fat free dry mass	WW%	15.22-22.71	18.75 **±** 2.40^ab^	16.24-23.76	18.58 **±** 2.30^ab^	13.55-20.53	17.02 **±** 1.80^b^	16.35-20.15	18.30 **±** 1.14^ab^	16.33-20.71	18.03 **±** 1.42^ab^	17.31-21.45	19.49 **±** 1.50^a^	0.086
	DW%	72.45-86.61	80.28 **±** 4.51	77.46-89.00	81.90 **±** 3.49	73.55-86.00	80.82 **±** 3.83	77.00-88.00	82.80 **±** 3.36	75.65-84.60	82.36 **±** 2.94	79.00-88.71	82.96 **±** 3.20	0.476

### Percent moisture influence on body constituents

Percent moisture showed highly significant correlation with body constituent in wet weight of *C. catla*; %Ash and fat contents in wet body weight have a highly significant positive correlation with percent moisture in T-O in control group, while non-significant with T-III, IV and V. Percent moisture (as independent factor) on dependent, a highly significant correlation (P<0.001) was observed with organic content and protein in all group except T-I in protein content (Table 2).

**Table 2:** Statistical parameters of %moisture content (%M) versus %body constituents in wet body weight (WW) of *C. catla.*

Correlation	Treatment	r	A	b	SE of b	*t*-Stat
%Ash(WW)=a + b %M	T-O	0.658***	71.200	1.377	0.557	0.039
	T-I	0.936***	98.385	−5.495	0.730	0.000
	T-II	0.014 ^ns^	78.764	0.040	0.991	0.969
	T-III	0.173 ^ns^	73.166	1.396	2.812	0.633
	T-IV	0.018 ^ns^	78.274	−0.043	0.871	0.962
	T-V	0.281 ^ns^	71.825	1.758	2.120	0.431
%Fat(WW)=a + b %M	T-O	0.644***	69.892	1.506	0.633	0.045
	T-I	0.497**	82.671	−1.304	0.805	0.144
	T-II	0.538**	83.715	−1.181	0.655	0.109
	T-III	0.285 ^ns^	79.416	−0.400	0.477	0.426
	T-IV	0.277 ^ns^	80.437	−0.603	0.741	0.439
	T-V	0.332 ^ns^	79.101	−0.649	0.652	−0.994
% Protein(WW)=a + b %M	T-O	0.947***	85.039	−0.564	0.068	0.000
	T-I	0.026 ^ns^	14.533	0.051	0.688	0.074
	T-II	0.815***	90.286	−0.873	0.220	0.004
	T-III	0.770***	88.698	−0.724	0.212	0.009
	T-IV	0.853***	90.079	−0.841	0.182	0.002
	T-V	0.817***	89.338	−0.763	0.190	0.004
% OC(WW)=a + b %M	T-O	0.965***	90.359	−0.708	0.069	0.000
	T-I	0.892***	−0.060	0.785	0.141	0.001
	T-II	0.942***	94.006	−0.884	0.111	0.000
	T-III	0.993***	95.963	−0.965	0.041	0.000
	T-IV	0.926***	93.725	−0.863	0.125	0.000
	T-V	0.989***	96.019	−0.937	0.049	0.000

%M = Percent moisture content; OC = organic content; r = Correlation Coefficient; a = Intercept; b = slope; S.E = Standard Error; *** P < 0.001; ** P < 0.01; ns = not significant (P > 0.05).

### Body constituents with body size (weight, length and condition factor)

Regression analyses among different parameters as percentage moisture, ash, fat, protein and organic content with wet body weight of *C. catla* respectively, are given in Table 3. Wet body weight with % moisture showed highly significant correlated (P<0.001) for T-O, I and T-V, least significant with T-III and non-significant T-II and T-IV; With percent ash (WW) showed highly significant correlation with all except T-III that showed non-significant correlation; with percent fat (WW) showed non-significant correlation with all except T-O as highly significant correlated; percent protein showed highly significant correlation with T-O, and T-I, significant with T-II, and III, least significant with T-V, while non-significant with T-IV; percent organic content showed highly significant correlation with T-O, and T-I, significant with T-II, least significant with T-III, while non-significant with T-IV and V.

**Table 3:** Regression analysis of wet body weight (WW, g) of fish with % body constituent in wet and dry weight of *C. catla.*

Equation	Treatment	R	a	B	S. E. (b)	*t* value when b = 0
%Moisture = a + b WW	T-O	0.678***	58.654	−0.639	0.245	0.031
	T-I	0.830***	−28.602	0.499	0.118	0.003
	T-II	0.339 ^ns^	21.467	−0.190	0.186	0.338
	T-III	0.411*	−32.404	0.531	0.417	0.238
	T-IV	0.327 ^ns^	−17.548	0.329	0.335	0.356
	T-V	0.816***	47.209	−0.484	0.515	0.004
%Ash (WW)=a + b WW	T-O	0.698***	15.141	−1.378	0.500	0.025
	T-I	0.873***	21.808	−3.080	0.610	0.001
	T-II	0.695***	10.001	−0.183	0.067	0.026
	T-III	0.285 ^ns^	−1.083	2.974	3.537	0.425
	T-IV	0.807***	15.692	−1.996	0.516	0.005
	T-V	0.291 ^ns^	17.575	−2.790	3.243	0.415
%Ash (DW)=a + b WW	T-O	0.719***	13.985	−0.250	0.085	0.019
	T-I	0.185 ^ns^	17.579	−0.448	0.841	0.608
	T-II	0.165 ^ns^	5.651	0.203	0.429	0.649
	T-III	0.453**	−0.794	0.637	0.443	0.189
	T-IV	0.633***	13.571	−0.314	0.136	0.049
	T-V	0.413*	17.646	−0.658	0.513	0.235
%Fat (WW)=a + b WW	T-O	0.937***	18.990	−2.068	0.273	0.000
	T-I	0.354 ^ns^	12.296	−0.559	0.522	0.315
	T-II	0.165 ^ns^	5.645	0.203	0.429	0.640
	T-III	0.115 ^ns^	8.196	0.209	0.639	0.752
	T-IV	0.199 ^ns^	6.434	0.435	0.758	0.582
	T-V	0.227 ^ns^	12.877	−0.682	1.033	0.528
%Fat (DW)=a + b WW	T-O	0.932***	16.619	−0.355	0.049	0.000
	T-I	0.014 ^ns^	9.882	0.006	0.160	0.970
	T-II	0.042 ^ns^	6.222	0.013	0.110	0.119
	T-III	0.230 ^ns^	7.334	0.096	0.144	0.523
	T-IV	0.318 ^ns^	5.333	0.157	0.165	0.369
	T-V	0.228 ^ns^	13.541	−6.254	1.358	0.532
%Protein (WW)=a + b WW	T-O	0.841***	2.648	0.473	0.107	0.002
	T-I	0.737***	18.803	−0.597	0.194	0.015
	T-II	0.509**	2.499	0.305	0.183	0.133
	T-III	0.497**	17.993	−0.603	0.373	0.144
	T-IV	0.089 ^ns^	9.373	0.088	0.348	0.806
	T-V	0.446*	0.613	0.639	0.453	0.006
%Protein (DW)=a + b WW	T-O	0.887***	−1.314	0.174	0.032	0.001
	T-I	0.019 ^ns^	9.470	0.008	0.148	0.958
	T-II	0.523**	−0.271	0.110	0.063	0.121
	T-III	0.360 ^ns^	19.000	−0.148	0.136	0.307
	T-IV	0.244 ^ns^	1.981	0.094	0.132	0.496
	T-V	0.541**	0.978	0.589	0.354	0..054
%OC (WW)=a + b WW	T-O	0.742***	−0.281	0.514	0.164	0.014
	T-I	0.809***	20.879	−0.577	0.148	0.005
	T-II	0.515**	1.853	0.271	0.159	0.127
	T-III	0.434*	19.200	−0.545	0.400	0.210
	T-IV	0.345 ^ns^	12.548	−1.545	0.354	0.321
	T-V	0.343 ^ns^	−0.232	0.498	0.482	0.332
%OC (DW)=a + b WW	T-O	0.719***	−10.975	0.250	0.085	0.019
	T-I	0.185 ^ns^	−27.266	0.448	0.841	0.608
	T-II	0.695***	−8.338	0.183	0.067	0.026
	T-III	0.453*	62.924	−0.637	0.443	0.189
	T-IV	0.365*	23.364	0.478	0.654	0.235
	T-V	0.413*	−48.162	0.658	0.513	0.235

OC = organic content; r = Correlation Coefficient; a = Intercept; b = slope; S.E = Standard Error; *** P < 0.001; ** P < 0.01; * P < 0.05; ns = not significant (P > 0.05).

Total length with % moisture showed highly significant correlated (P<0.001) with T-I, least significant with T-II and non-significant T-O, T-III, T-IV and T-V; percent ash (WW) showed highly significant correlation with all except T-O, T-III that showed non-significant correlation and least significant with T-V; percent fat (WW) showed non-significant correlation with all groups; percent protein showed highly significant correlation with T-I, and T-II, significant with T-V, least significant with T-III, while non-significant with T-O and T-IV; percent organic content showed highly significant correlation with T-I, and T-II, least significant with T-V, while non-significant with T-O, T-III and IV. Condition factor showed least significantly correlated with percentage moisture in T-O; in T-O of ash showed highly significant correlation; significant with fat in T-O;T-O significant, T-II least significant for percent protein and significant for T-O, least significant with T-III for organic content indicated in Table 4.

**Table 4:** Regression analysis of total length (TL) of fish with % body constituent in wet (WW) and dry body weight (DW) of *C. catla.*

Equation	Treatment	r	a	b	S. E. (b)	*t* value when b = 0
%Moisture = a + b TL	T-O	0.271 ^ns^	16.345	−0.080	0.100	0.448
	T-I	0.633***	−5.679	0.202	0.087	0.049
	T-II	0.404*	17.555	−0.116	0.093	0.247
	T-III	0.293 ^ns^	0.265	0.125	0.144	0.411
	T-IV	0.233 ^ns^	0.918	0.108	0.160	0.517
	T-V	0.382 ^ns^	19.976	−0.130	0.111	0.276
%Ash (WW)=a + b TL	T-O	0.162 ^ns^	9.849	0.099	0.214	0.655
	T-I	0.742***	15.216	−1.386	0.443	0.014
	T-II	0.740***	10.806	−0.593	0.191	0.014
	T-III	0.277 ^ns^	6.787	0.953	1.170	0.439
	T-IV	0.773***	12.716	−0.884	0.256	0.009
	T-V	0.390*	12.228	−0.829	0.693	0.266
%Ash (DW)=a + b TL	T-O	0.048 ^ns^	10.155	0.005	0.038	0.895
	T-I	0.369*	17.879	−0.472	0.421	0.295
	T-II	0.840***	10.606	−0.113	0.026	0.002
	T-III	0.374*	7.348	0.174	0.152	0.287
	T-IV	0.354 ^ns^	3.547	0.234	0.221	0.341
	T-V	0.347 ^ns^	11.254	−0.578	0.954	0.465
%F (WW)=a + b TL	T-O	0.232 ^ns^	10.966	−0.159	0.236	0.519
	T-I	0.150 ^ns^	10.414	−0.125	0.292	0.680
	T-II	0.028 ^ns^	8.358	0.018	0.222	0.938
	T-III	0.144 ^ns^	9.686	0.087	0.210	0.691
	T-IV	0.352 ^ns^	7.941	0.080	0.076	0.319
	T-V	0.307 ^ns^	10.840	−0.205	0.224	0.388
%Fat (DW)=a + b TL	T-O	0.284 ^ns^	10.908	−0.034	0.040	0.426
	T-I	0.157 ^ns^	9.220	0.038	0.084	0.666
	T-II	0.143 ^ns^	8.866	−0.023	0.056	0.693
	T-III	0.222 ^ns^	0.987	0.031	0.048	0.530
	T-IV	0.343 ^ns^	7.923	0.080	0.176	0.311
	T-V	0.469*	11.351	−0.078	0.052	0.171
%Protien (WW)=a + b TL	T-O	0.175 ^ns^	9.796	0.030	0.061	0.629
	T-I	0.606***	13.735	−0.260	0.121	0.063
	T-II	0.702***	5.628	0.215	0.077	0.024
	T-III	0.404*	12.434	−0.162	0.130	0.246
	T-IV	0.040 ^ns^	9.617	−0.018	0.162	0.914
	T-V	0.561**	7.016	0.178	0.093	0.091
%Protein (DW)=a + b TL	T-O	0.119 ^ns^	9.789	0.007	0.021	0.743
	T-I	0.081 ^ns^	11.069	−0.018	0.078	0.824
	T-II	0.763***	3.411	0.082	0.024	0.010
	T-III	0.329 ^ns^	13.035	−0.045	0.045	0.354
	T-IV	0.054 ^ns^	1.2145	−0.458	0.125	0.659
	T-V	0.531**	5.285	0.066	0.037	0.114
%OC (WW)=a + b TL	T-O	0.143 ^ns^	9.656	0.031	0.075	0.694
	T-I	0.602***	14.183	−0.227	0.107	0.066
	T-II	0.626***	5.561	0.168	0.074	0.053
	T-III	0.331 ^ns^	11.458	0.045	0.047	0.325
	T-IV	0.121 ^ns^	8.541	0.025	0.365	0.784
	T-V	0.421*	7.193	0.136	0.103	0.226
%OC (DW)=a + b TL	T-O	0.048 ^ns^	10.674	−0.005	0.038	0.895
	T-I	0.369*	−29.311	0.472	0.421	1.122
	T-II	0.840***	−0.705	0.113	0.026	0.002
	T-III	0.329 ^ns^	13.035	−0.045	0.045	0.354
	T-IV	0.347 ^ns^	12.254	−0.254	0.874	0.398
	T-V	0.513**	−6.052	0.181	0.107	0.130

OC = organic content; r = Correlation Coefficient; a = Intercept; b = slope; S.E = Standard Error; *** P < 0.001; ** P < 0.01; * P < 0.05; ns = not significant (P > 0.05).

### Digestive enzyme

In present analysis, maximum value of relative gut mass was observed in T_-_V with maximum mean value 0.078 ± 0.013 IU/ml.mn^-1^, Relative gut length and relative gut length found maximum in T-V with mean value 0.078 ± 0.013 IU/ml.mn^-1^, and 7.86 ± 0.09 IU/ml.mn^-1^ as similar trend was observed for Zihler’s and Gastrosomatic index (GSI) (Table 5).

**Table 5:** Mean±S.D values of fish size and gut morphometric indices of Relative gut mass, Relative gut length, Zihler’s index, and Gastrosomatic index under different concentration in *C. catla.*

Feeding groups	N	Fish Size		Gut morphometric indices					
		Body Weight (g)	Total Length (cm)	Relative Gut Mass (g)		Relative Gut Length (cm)		Zihler’s GSI/Gastrosomatic index Index (ZI)	
				Mean. ± S.D	Range	Mean±S.D	Range	Mean. ± S.D	Range
**T-O**	10	10.34 ± 0.73	10.69 ± 0.75	0.071 ± 0.003	0.069 to 0.076	6.12 ± 0.45	6.01-6.55	7.54 ± 3.39	7.21 ± 0.49
**T-I**	10	9.43 ± 0.46	8.24 ± 1.80	0.068 ± 0.003	0.062 to 0.075	7.37 ± 0.07	7.27 to 7.47	3.62 ± 0.28	6.92 ± 0.45
**T-II**	10	6.10 ± 1.87	8.15 ± 1.04	0.073 ± 0.011	0.070-0.088	6.22 ± 0.21	6.25-6.76	8.07 ± 0.86	7.65 ± 0.17
**T-III**	10	6.95 ± 1.85	8.74 ± 0.88	0.075 ± 0.012	0.071-0.081	6.33 ± 0.22	6.21-6.69	8.07 ± 0.86	7.65 ± 0.17
**T-IV**	10	9.74 ± 2.41	10 ± 0.70	0.072 ± 0.011	0.061-0.093	7.23 ± 0.31	7.11-7.33	4.45 ± 1.18	7.04 ± 0.66
**T-V**	10	9.43 ± 0.98	8.24 ± 2.92	0.078 ± 0.013	0.069-0.092	7.86 ± 0.09	7.79 to 7.96	5.24 ± 0.88	7.46 ± 0.33

**Fig 2:**
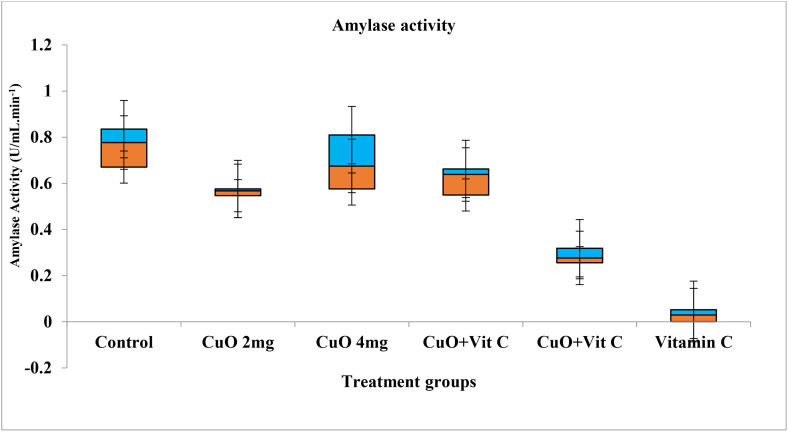
Amylase activity exposed to different concentration in *C. catla.*

**Fig 3:**
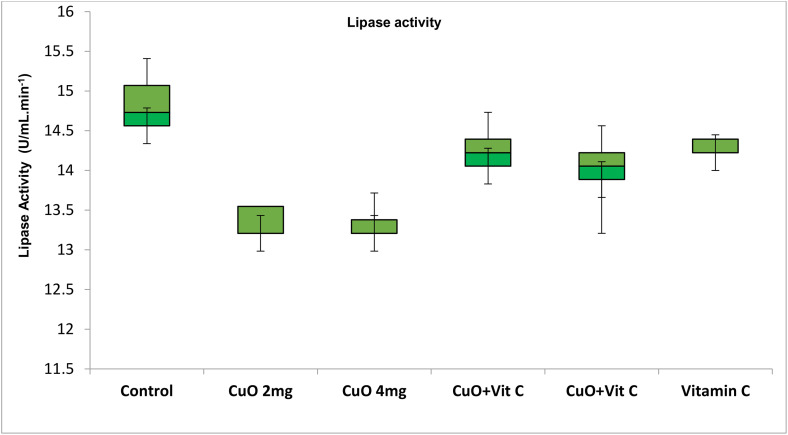
Lipase activity exposed to different concentration in *C. catla.*

**Fig 4:**
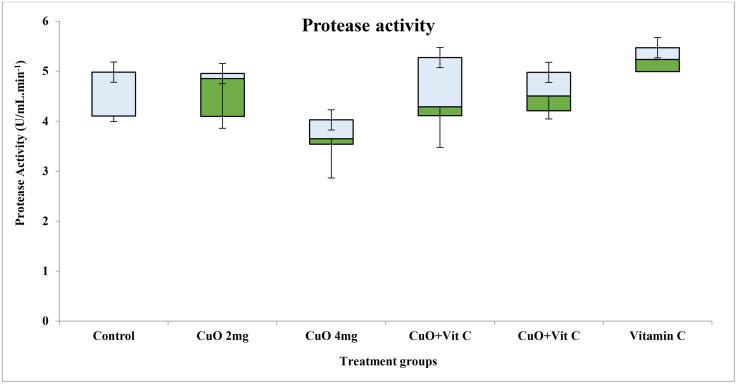
Protease activity exposed to different concentration in *C. catla.*

Activities of digestive enzymes in various groups showed a significant difference for different concentrations. Maximum value of protease activity was observed in T-V 5.734 ± 0.60 (U/mL.min^-1^) with range 4.99–6.60, while lower in T-II (4.68 ± 0.78) (Fig 2). Activities of amylase was found maximum value in T-V 0.74 ± 0.14 (U/mL.min^-1^) with range 0.91–0.56, while lower in T-V (0.54 ± 0.19) (Fig 3). Maximum activities of lipase activity was observed in T-0, while lower in T-II (Fig 4).

### Elemental concentration

Results for elemental concentration of *Catla catla* exposed to the nanoparticle (CuO-NPs) and Vitamin C are given in Table 6. Results shows that Cd, Pb and Co were not quantified in the fishes of any experimental groups. Macroelements (K and Na) were not found significantly different (P > 0.05) among different studied groups. Fe concentration was found the highest in T-V. Zn concentration was also found the highest in T-V, which was not significantly different (P > 0.05) with the *C. catla*, reared in T-O. While, concentrations of Cr and Cu were found significantly higher in T-II, where the fishes were fed a diet containing 4 mg/kg CuO-NPs.

**Table 6:** Mean values±S.D. of metal concentrations (µg/g) in wet weight of *C. catla* reared in different treatments.

Elements	T-O (Control)		T-I (CuO 2 mg)		T-II (CuO 4 mg)		T-III (CuO 2 mg + Vit 250 mg)		T-IV (CuO 4 mg + Vit 250 mg)		T-V (Vit 250 mg)		Sig.
	Range	Mean±S.D.	Range	Mean±S.D.	Range	Mean±S.D.		Range	Mean±S.D.	Range	Mean±S.D.	Range	Mean±S.D.
**Cd**	NQ	NQ	NQ	NQ	NQ	NQ	NQ	NQ	NQ	NQ	NQ	NQ	--
**Fe**	5.00 to 14.01	10.41 **±** 2.95^ab^	7.71 to 11.70	9.34 **±** 1.35^bc^	4.49 to 9.64	7.60 **±** 1.54^c^	6.34 to 14.72	10.63 **±** 2.81^ab^	7.57 to 15.21	10.71 **±** 2.26^ab^	6.67 to 15.44	12.63 **±** 2.99^a^	0.001
**K**	328.63 to 731.17	528.74 **±** 120.89	259.04 to 578.59	377.25 **±** 86.49	321.12 to 636.56	453.61 **±** 90.50	293.57 to 642.91	449.97 **±** 118.85	348to 646.83	476.1 **±** 94.06	361.13 to 817.88	515.93 **±** 174.02	0.076
**Na**	132.53 to 377.93	208.79 **±** 83.70	69.43 to 230.24	160.51 **±** 51.21	76.44 to 205.44	137.7 **±** 41.14	100.54 to 247.47	166.45 **±** 47.76	115.79 to 250	186.15 **±** 48.46	106.21 to 287.14	175.25 **±** 62.84	0.139
**Pb**	NQ	NQ	NQ	NQ	NQ	NQ	NQ	NQ	NQ	NQ	NQ	NQ	--
**Co**	NQ	NQ	NQ	NQ	NQ	NQ	NQ	NQ	NQ	NQ	NQ	NQ	--
**Zn**	6.79 to 22.35	10.85 **±** 4.67^a^	2.34 to 8.75	5.84 **±** 1.89^b^	2.83 to 6.67	4.84 **±** 1.20^b^	1.67 to 10.76	4.34 **±** 3.16^b^	1.58 to 8.02	5.10 **±** 2.03^b^	8.79 to 16.23	12.59 **±** 2.10^a^	0.000
**Cr**	0.00 to 0.01	0.01 **±** 0.002^b^	0.00 to 0.02	0.01 **±** 0.005^b^	0.00 to 0.005	0.02 **±** 0.02^a^	0.00 to 0.02	0.01 **±** 0.005^b^	0.00 to 0.01	0.01 **±** 0.001^b^	0.00 to 0.01	0.01 **±** 0.001^b^	0.006
**Cu**	0.05 to 0.70	0.23 **±** 0.18^d^	0.13 to 2.78	1.55 **±** 0.79^c^	1.76 to 5.40	3.72 **±** 0.97^a^	0.13 to 2.54	1.41 **±** 0.72^c^	1.79 to 3.53	2.43 **±** 0.56^b^	0.01 to 0.47	0.14 ± 0.15^d^	0.000

ANOVA and regression analysis to examine how different treatments affected the amounts of different elements (Fe, K, Na, Zn, and Cu) in distinct groups. T-I (CuO-NPs 2 mg/kg), T-II (CuO-NPs 4 mg/kg), T-III (CuO-NPs 2 mg/kg + Vitamin C 250 mg/kg), T-IV (CuO-NPs 4 mg/kg + Vitamin C 250 mg/kg), and T-V (Vitamin C 250 mg/kg) were the treatment groups. Significant differences (p < 0.05) were found in the ANOVA findings for every element across the groups, indicating that the treatments had different effects on elemental concentrations. To be more precise, the levels of Fe, K, Na, and Zn were typically lower in CuO-NPs treatments (T-I, T-II) when combined with Vitamin C (T-III, T-IV), but the levels were frequently restored to levels that were either higher or comparable to those of control group. Its preventive or boosting impact was highlighted by the fact that vitamin C alone (T-V) showed the bit higher concentrations for the majority of elements. These results were corroborated by regression analysis, which revealed significant interaction terms between CuO-NPs and Vitamin C, illuminating Vitamin C moderating effects on CuO-NPs induced elemental disruptions (Table 7).

**Table 7:** Regression analysis of various treatment groups of *C. catla.*

Element	Variable	Coefficient	Std. Error	t-Statistic	P-Value
Fe	T-0	10.410	2.760	3.770	0.0003
	T-I	−2.110	1.850	−1.140	0.260
	T-II	−3.430	1.540	−2.230	0.030
	T-III	2.200	5.760	0.380	0.700
	T-IV	−1.190	2.930	−0.410	0.680
	T-V	4.130	4.030	1.020	0.320
K	T-0	528.740	116.62	4.530	0.0001
	T-I	−117.520	92.210	−1.270	0.210
	T-II	−135.970	71.550	−1.900	0.060
	T-III	−1.800	112.690	−0.020	0.990
	T-IV	−61.710	177.340	−0.350	0.730
	T-V	168.280	151.290	1.110	0.270
Na	T-0	208.630	81.240	2.570	0.020
	T-I	−108.130	24.510	−4.410	0.0004
	T-II	−121.380	30.870	−3.930	0.0010
	T-III	−18.010	96.300	−0.190	0.850
	T-IV	−40.860	60.910	−0.670	0.510
	T-V	13.110	99.850	0.130	0.900
Zn	T-0	10.850	4.920	2.210	0.040
	T-I	−6.270	2.650	−2.370	0.030
	T-II	−7.750	1.180	−6.570	0.0002
	T-III	−4.910	3.490	−1.410	0.180
	T-IV	−5.770	2.740	−2.110	0.040
	T-V	0.80	4.420	0.180	0.860
Cu	T-0	1.010	0.940	1.070	0.290
	T-I	−0.780	0.180	−4.330	0.0005
	T-II	3.040	1.590	1.910	0.060
	T-III	1.720	3.320	0.520	0.600
	T-IV	0.130	0.680	0.190	0.850
	T-V	−0.240	0.300	−0.800	0.430

## Discussion

The present study is one of the first reports detailing the effects of Cu-NPs compared to Copper added. Chen et al. [17] reported that the reduction in growth performance was most likely due to two reasons: first, Copper exposure caused increased metabolic expenditure for detoxification and maintenance of homeostasis; second, higher Copper exposure reduced feed intake. Indeed, growth is a complex phenomenon that partly relies on the digestive capabilities of an organism [18]. Sherwood et al. [19] reported that yellow perch living in lakes subjected to chronic exposure Cu-NPs.

In present study body composition analysis of *C. catla* exposed to copper oxide nanoparticle revealed maximum protein content with vitamin concentration, an increase in nanoparticle concentration inverse effect in carcass protein was observed, though it showed non-significant differences. Moisture contents also showed variations in proteins, ash and fat showed significant differences. Our findings are as similar as observed by Umer and Ali [20] because they also observed non-significant variations in moisture and protein contents and significant variations in ash and fat contents. However, Rigos et al. [21] and Mongile et al. [22] observed non-significant variations in moisture and fat contents. Considering body composition, protein contents in experimental groups remained almost stable as showed lower protein contents. Total organic contents also showed non-significant variation [23]. Storebakken and Austreng [24] assumed that appropriate dietary protein level has an influence on fat content and reflects variation in fat deposition in fish body. Furthermore, these result revealed that protein in fish does not readdress the composition of diet. The nutrient level as well as anti-nutritional level, diet acceptability, diet palatability, fish behavior and feed preference may also influence on body composition [25]. A slight variation in body composition were observed by Khan et al. [5]. The change in feeding habit, feed formula and nutrients level during growth may also have an influence on muscle fat and moisture contents ratio [26]. In our findings; moisture concentration is higher in all the experimental groups with low organic content values. Thus, fish is non-fatty with loss of muscle proteins. It is observed that when fish is fed with higher proportion of lipids; its body shows an apparent decrease in protein contents. It is an agreement with our finding.

Chen et al. [17] reported that deposition of lipids was influenced by several factors, but there was a general trend for percentage body lipids to decrease with decreasing fish size, and any decrease in the percentage of lipids was usually accompanied by an increase in percentage of body moisture content. The results of the present study are in general accord with this. It is likely that the proteins and lipids in fish can be used as energy source for detoxification and the maintenance of homeostasis during metal exposure [27]. In the present study, crude proteins and crude lipids decreased with an increase Copper-NPs dose, indicating that Cu ions were more harmful to energy stores (such as crude proteins and crude lipids) and weight gain than Cu-NPs. However, Chen et al. [17] found increased lipid content of yellow catfish as in general agreement with the present analysis.

Present study revealed that wet body weight and total length of fish has a positive significant correlation with proteins, fats, ash and moisture contents. Condition factor have an impact on body composition [14], increase in condition factor results in decrease of moisture contents. A significant and highly significant correlation was observed between fat contents and condition factor of fish which is similar to the findings of Yousaf et al. [28]. Protein contents when plotted against condition factor, a positive significant correlation was observed. Higher is the protein concentration in the body of fish higher is the condition factor and reverse is the moisture concentration [29]. However, sometime problem arises in interpretation of these parameters because weight gain by fish is not always proportional to the cube of its length [30] The weight-length relationship and condition factor are widely used indicators of growth efficiency and overall well-being in fish populations, contributing to effective stock assessment and management practices [31–33]. When a fish is not fed with sufficient feed, it may also show variation in its condition factor. Grow out fish is usually more selendrical and have low condition factor as compared to brooder fish [34]. Additionally, variations in proximate composition, influenced by body size and physiological status, play a significant role in determining the nutritional value and market quality of fish [35–44].

In recent decade, fish meal substitutes have been reported by many nutritionists in fish feed to make this area cost effective and more productive; furthermore, digestive secretion at various feed inputs were also studied to optimize the diet compositions [45]. Different feed inputs in aquaculture practices affect the digestive activity of fish, because it modulates their digestive mechanism. RGL indices in our study exhibited positive correlation with fish body weight and length. But, Day et al. [46] confirmed a negative correlation of gut variables to fish body size.

Digestive enzyme activity (e.g., protease, amylase, and lipase) can be used as an indicator of potential feed utilization and growth differences [18] and to some extent may serve as an indicator of the digestive capacity in relation to the type of feed offered and the properties of aquaculture environments [47]. In our study, the activities of protease, amylase, and lipase decreased with increasing Cu-NPs dose, suggesting that either Cu-NPs or exposure decreased digestive capability.

RGM, DSI and ZI are the potential gut morphometric indices used to explore and identify fish feeding habits, primarily associated with gut length. Zihler [48], Kramer and Bryant [49] categorized the fish on the basis of ZI into three categories; 0.3 to 3.0 (carnivorous), 2.4 to 5.8 (omnivorous) and 11.6 to 55.0 ZI value (herbivorous).

Hani et al. [10] noted similar results in sticklebacks, depicting that small fish increases their gut mass to maximize absorption rate and extraction of nutrients to generate maximum energy to ensure growth. Thus, increased gut mass ensures maximum intake of nutrients which directly relates to increased energy intake from food [50]. Condition factor (K) is an indicator of fish health status, it also have positive influence on digestive enzyme and fish visceral components. Maximum condition factor shows less RGM and ZI representing maximum growth and ideal health status. Previously no such information was expressed in literature, so further investigation is required. Melo et al. [51] showed reverse increment order in amylase activities. Ismat et al. [52] observed that in Indian major carps; amylase activities have not been influenced by rice police, soybean meal and cotton seed meal.

Protease activity in gut increases with the increase in carbohydrate level. Rodiles et al. [22] studied the significant influence of protein inputs on protease secretions of sole (*Solea senegalensis*). Lipase activity was observed highest, a gradual increase in lipase activity was noted with the increment. Su et al. [53] showed that dietary Chitosan oligosaccharide (COS) significantly proliferate growth performance, DSI, intestinal protease as well as lipase activity in *Takifugu rubripes*.

Contamination of fish food with various chemical has turned into a global concern. Even though fish is considered as a high quality protein source, but in wild the population of fish may have higher concentration of heavy metals. So, it is used as good indicators to monitor the contamination. Excessive accumulation of metal in fish body is not good it cause toxicity. There is increases in accumulation level of these metals day by day and can’t easily digested in human body [54]. As accumulation of these metals in fish body to risky levels has turn issues and increases health concern. Gills are an important organ for both osmoregulation and respiratory gas exchange, and they were the primary target for toxicity of Cu-NPs [55]. In the present study, gills had areas of hyperplasia at the base of the secondary lamellae. These results were similar with the data recorded by Al-Bairuty et al. [56] and Griffitt et al. [55].

The concentration of calcium found highest and Cadium with lowest concentration as studied by Naeem et al*.* [57] in *O. mykiss*; Naeem et al*.* [15] in *Aristichthys nobilis* and Khalid et al*.* [58] in *C. idella.* Iron plays physiological role in living being, if concentration is in access than it causes harmful effects [59]. Access concentration of Iron in fish did is harmful for fish acclimation and feed conversion efficiency of fish as it influences the growth [60]. Concentration of Iron (Fe) was found under permissible limit as proposed by WHO and FAO, as cited in study by Kundu et al. [61] as 80 μgg^-1^. Naeem et al*.* [15] studies reported that the concentration of iron 61.64 ± 6.21 in *A. nobilis* and 38.57 ± 1.758 in *O. mykiss* [57] as found close to the present study.

Concentration of Fe varied between 5.00 and 14.01 µgg-1, 2.85 µgg-1 in *H. fossilis* and 669.0 µgg-1 for *C. catla* [62]. As our study concentration is found in between these values. Fe in wet and dry body weight reported by Yousaf et al. [63] very similar to the present study. Maximum permissible limit for Fe as FAO/ WHO [64] is 100 µgg-1 as indicates that in present study Fe is under maximum permissible limit.

The mean copper level in the control group (T-O) was 1.01 ± 0.94 µgg-1, with a range of 0.13 to 2.78 µgg-1. With a mean of 0.23 ± 0.18 µgg-1, CuO-NPs treatment at 2 mg/kg (T-I) considerably decreased copper levels, whereas treatment at 4 mg/kg (T-II) significantly elevated copper levels with a mean of 4.05 ± 1.59 µgg-1. Vitamin C was used in conjunction with T-III and T-IV to modify these effects. These studied values were found under maximum permissible limit by FAO/WHO [64]. Similar concentration of copper was already reported in grass carp as 8.91 ± 1.17 µgg-1 in dry weight and 1.81 ± 0.23 µgg-1 in wet weight by Khalid et al. [58] reported the similar values.

Zinc levels in the T-O control group varied between 6.79 and 22.35 µgg^-1^, with an average of 10.85 ± 4.92 µgg-1. Zinc levels were decreased by CuO-NPs treatments alone (T-I and T-II), with averages of 4.58 ± 2.65 µgg-1 and 3.10 ± 1.18 µgg^-1^, respectively. Zn is trace element that is essential for transfer of electron in different catalytic reactions. While high intake causes bioaccumulation in body organism that caused toxicity [65]. Zn is important for gene expression, cell based growth and for different metallo-enzymes [66]. It is observed that the concentration of Zinc (Zn) in present study found in normal range as recommended by WHO/FAO [64]. The effect of varying diet levels was evaluated on composition under T1 to T6 level [67], Result found very close with the study by Naeem et al. [57] with mean value of 15.49 ± 1.41 in farmed *A. nobilis;* 16.96 μgg^-1^ in *M. bleekeri* by Naeem et al. [15]. Study reported the concentration 39.7 µgg-1 in *O. niloticus.* As this is also very close to our study. Cd was not quantifiable in *O. niloticus* [68] and wild *O. niloticus* [15]*.* Observations found similar with present study results. Co is also not detected in study, although reported concentrations found under permissible limit as 0.1 μgg^-1^ [69].

## Conclusion

Growth performance of *C. catla* found T-V (Vitamin C) showed that Percent moisture content lowest mean value of percent moisture content was found in T-V, while highest value in T-II. As with highest protein content in T-V and lowest in T-II. Activities of digestive enzymes in various groups showed a significant difference with maximum value of protease activity in T-V, while lower in T-II. Activities of amylase was found maximum value in T-V, while lower in T-V. Maximum activities of lipase activity was observed in T-O, while lower in T-II. Concentration of Cadmium, lead and cobalt was non-quantifiable among all treatment groups, while all other metal values found below the detection limit, which revealed significant interaction terms between CuO and Vitamin C, illuminating Vitamin C moderating effects in all parameters.
